# Decoding stillbirths using the Relevant Condition at Death classification: Study from the developing world

**DOI:** 10.4274/jtgga.galenos.2018.2018.0080

**Published:** 2019-05-28

**Authors:** Neeraj Kulkarni, Deepti Pinto Rosario, Liji Sarah David, Reeta Vijayaselvi, Manisha Madhai Beck

**Affiliations:** 1 Department of Obstetrics and Gynecology, Christian Medical College, Vellore, India; 2 Head of Obstetrics and Gynecology, Christian Medical College, Vellore, India

**Keywords:** Stillbirth, ReCoDe intrauterine fetal demise, developing world, gestational hypertension, uteroplacental insufficiency

## Abstract

**Objective:**

To determine the stillbirth rate in 2017 at Christian Medical College, a tertiary care perinatal center in South India, and to identify causes for the various stillbirths that occurred using the Relevant Condition at Death (ReCoDe) classification.

**Material and Methods:**

Medical records of the women with stillbirths between January 1^st^, to December 31^st^, 2017, were retrieved and analyzed using the SPSS software (IBM, version 23). The study was approved by the institutional review board (minute no: 11273, retro dated: 28/3/2018).

**Results:**

Of the total 14696 deliveries between January 1^st^, 2017, to December 31^st^, 2017, there were 247 stillbirths, a rate of 16.8 per 1000 births. Maternal factors: 156 (64.2%) women were booked and the rest were un-booked. Hypertensive disorders of pregnancy were detected in 27.5% (n=67). A greater number of un-booked women had gestational hypertension as compared with booked women (41% vs 24%, p=0.005). Fetal characteristics: still births secondary to lethal congenital anomalies were seen in 18.2% (n=45). Lethal congenital anomalies were diagnosed 10 times more in the booked patients than un-booked ones (24.7% vs 2.3%, p=0.001). Obstetric factors: one or two previous miscarriages were seen in 29.5% cases. Seventeen women (6.9%) had a prior stillbirth. ReCoDe Classification: we were able to successfully classify 84.2% of the stillbirths, leaving 15.78% unclassified. Fetal growth restriction secondary to uteroplacental insufficiency was found in 25.9% cases. Of the placental causes, abruption accounted for 10.9% of cases. Medical co-morbidities were seen in 46.5% pregnancies.

**Conclusion:**

The ReCoDe method of classifying stillbirths is useful in the developing world. It helped to elucidate the cause for stillbirths in 84.2% of cases. The majority of cases in our set were due to fetal growth restriction, hypertensive disorders of pregnancy, and uteroplacental insufficiency. Stillbirths can be prevented by a comprehensive antenatal care system, early recognition, and close monitoring of high-risk pregnancies.

## Introduction

The World Health Organization (WHO) defines stillbirth as the delivery of a fetus after 22 completed weeks of gestation, weighing 500 grams or more, with the newborn showing no signs of life at delivery (1). According to the WHO, there were 2.6 million stillbirths in 2015. One out of every 45 babies was stillborn. Nearly three-quarters of them were from South Asia and sub Saharan Africa. The stillbirth rate in India was 23/1000 births in 2015, compared to a worldwide rate of 18.4/1000 births (2). 

Since then, the stillbirth rate in our country has declined by 10%, with an annual reduction rate of 2% between 2000-2015. This decline, however, is slow in comparison to the annual reduction in maternal mortality rate and under 5 infant mortality rate at 3% and 3.9%, respectively, during the same period (2). The WHO targets reducing the stillbirth rate to 12/1000 by 2030 by adopting the “Every newborn action plan” (2). 

Socioeconomic factors and lack of appropriate antenatal care both play major roles in the occurrence of stillbirths. However, there are still lacunae in the knowledge of biomedical causes of stillbirth due to a lack of clear, reliable data from the developing world, including India. Stillbirths are traumatic to both the parents and the treating obstetrician. Often there is an element of stigma attached to giving birth to a stillborn baby and the mother holds herself responsible. Elucidating a cause for stillbirth, therefore, becomes quite challenging because parents do not often consent for diagnostic tests such as autopsy or placental biopsy. Hence, the majority of stillbirths remain “unexplained” because a complete evaluation cannot be undertaken.

Although the majority of stillbirths occur in South Asia and Sub Saharan Africa, there are surprisingly few studies on stillbirths from these countries. There is a wide research gap that needs to be filled before the stillbirth rate can be lowered to the desired level.

Relevant Condition at Death (ReCoDe) is a classification derived from a population-based cohort study in West Midlands Perinatal Institute, England. Unlike older classifications, ReCoDe seeks to identify the relevant condition at the time of fetal death – “What went wrong, not necessarily why” (3). It is able to classify nearly 85% of stillbirths, unlike older classifications, which left 50-66% of cases as unclassified (4). It is structured in the form of a hierarchy, starting from conditions that affect the fetus and moving outwards in simple anatomic groups. These are subdivided into pathophysiologic conditions where the primary condition that is applicable to a case, should be first on the list (3). As this classification is more reliant on clinical information and not autopsy/histopathologic data, it has relevance in the developing world where autopsy and/or placental biopsy is not routinely performed due to a lack of expertise and unwilling parents.

Therefore, we performed this study to determine the stillbirth rate over the period of one year at Christian Medical College, a tertiary care perinatal center in South India, and to identify causes for the various stillbirths that occurred using the ReCoDe classification.

### Operational definitions

**Stillbirth:** Delivery of a fetus after 22 completed weeks of gestation, weighing 500 g or more with the newborn showing no signs of life after delivery (5).

**Hypertensive disorders of pregnancy include:** Pre-eclampsia/eclampsia/syndrome of Hemolysis; Elevated Liver enzymes, Low Platelets (HELLP)/chronic hypertension with superimposed pre-eclampsia (6).

**Medical disorders:** Including diabetes mellitus, chronic hypertension, autoimmune conditions, anti-phospholipid antibody (APLA) syndrome (7).

**Uteroplacental insufficiency:** The presence of one or more clinical indicators of maternal vascular malperfusion such as fetal growth restriction; oligohydramnios and abnormal pulsed-flow Doppler studies (8).

**Fetal growth restriction: **Estimated fetal weight (EFW) measured on scans using Hadlock’s formula, less than the 10^th^ percentile based on WHO sex-specific growth charts (9).

**Induction of labor: **Stimulation of artificial uterine contractions before the onset of labor, with or without ruptured membranes (10). Performed in cases where continuation of pregnancy has potential threat to life of mother and/or baby. May also be performed if the fetus has died or has severe abnormality.

## Material and Methods

The hospital numbers of the mothers who gave birth to stillborn babies between January 1^st^, 2017 to December 31^st^, 2017, were retrieved from the electronic birth registry maintained by trained nurses. Medical records were then retrieved and data was reviewed by a panel consisting of two senior consultant obstetricians with over fifteen years’ individual clinical experience, and two junior consultant obstetricians (with 5 years’ individual clinical experience). In the event of any dissent, the records were analyzed by a third senior obstetric consultant, who was blinded to the opinion of the others on the panel, for the final diagnosis. The diagnosis of congenital anomalies on antenatal scans was made by the Fetal Medicine Foundation (FMF) (United Kingdom accredited sonographer in all cases). Information on twin pregnancies was also included. Time of death of one/both twins was noted, and cause of death was investigated.

### Ethics committee approval

The study was approved by the Institutional Review Board of the hospital (minute no: 11273, retro dated: 28.03.2018). Consent was not taken, given the retrospective nature of the study and anonymous data collection.

### Statistical analysis

Data analysis was performed using SPSS software (IBM, version 23). Descriptive measures such as mean, median, and standard deviation were computed for all continuous variables. Frequency data and cross tables were compared using the chi-square test as appropriate. For all statistical tests, p values <0.05 were considered as statistically significant.

## Results

Of the total 14696 deliveries between January 1^st^, 2017, to  December 31^st^, 2017, there were 243 deliveries, which resulted in 247 stillbirths, a stillbirth rate of 16.8 per 1000 births. 

### Maternal characteristics

There were 202 (83.1%) mothers from local areas and 41 mothers (16.9%) from other parts of India. Whereas 64.2% (n=156) were booked at our hospital with regular antenatal check-ups since early pregnancy, 35.8% were referred for the first time to our hospital at the time of diagnosis of intrauterine demise in the antepartum or intrapartum period. The majority (75%) of the pregnant women were aged between 21-30 years. Around 9% of the women were aged below 21 years (Table 1). There was no particular association between any subgroup of stillbirths and particular age group of patients.

A body mass index (BMI) of >25 kg/m^2^ was seen in 117 (48.1%) of women who had stillbirth. Only 21 (8.6%) patients were underweight. There was no significant correlation, however, between BMI and any particular etiology of stillbirth such as congenital anomalies or hypertensive disorders. Primiparous women constituted 59.3% (n=144) of the study population, whereas 40.7% (n=99) were multiparous women. The majority of the women underwent vaginal delivery (88.9%, n=216). Twenty-five (10.3%) needed lower segment cesarean section (LSCS), and two (0.8%) of them underwent operative vaginal delivery (Table 1).

Of the 25 patients who had a previous LSCS, 60% (n=15) had a vaginal delivery and 40% (n=10) had a repeat surgery (Table 2).

### Obstetric risk factors

Sixteen (6.6%) patients had history of sub-fertility and conceived with the help of artificial reproductive techniques (Table 3). Fifty-five (22.6%) patients had one miscarriage in the past, and 17 (6.9%) patients who had two miscarriages. There were 17 (6.9%) patients who had a prior stillbirth. Ten out of the 17 stillborn babies were diagnosed as having hydrops, requiring induction of labor. The majority (9/10) had non-immune hydrops. Half of the six women with bicornuate uterus had placental abruption leading to stillbirth. Ten percent (n=25) had a history of previous LSCS. 

### Medical risk factors

Associated medical conditions were present in 46.5% (n=113 pregnancies), Table 4. These included chronic hypertension, diabetes mellitus, APLA, systemic lupus erythematosus, and obstetric cholestasis. The most common medical condition was diabetes mellitus, seen in 59.2% (67/113) of cases. Most women with medical disorders had at least two comorbidities, the most common combination being chronic hypertension and diabetes mellitus.

Hypertensive disorders of pregnancy were found in 54.8% (62/113) pregnancies. The incidence of severe hypertensive disorders (severe pre-eclampsia/eclampsia/HELLP syndrome) among booked patients was almost half of that seen among the un-booked patients (24% vs 41%, p=0.005) (Table 4). 

The majority of stillbirths occurred in the third trimester (n=161, 66.2%). The majority of stillbirths in the third trimester were due to fetal growth restriction. Across all gestations, the majority of patients were booked. In pregnancies <28 weeks, 74.3% were booked patients, whilst booked patients made up 67.4% of patients in term pregnancies (Table 5).

### Induction of labor

There were 148 (60.90%) pregnancies that underwent induction of labor. Most of these cases were induced with prostaglandin E1, and the remaining with intravenous oxytocin. The indications are summarized in Table 6.

Intrauterine death (IUD) was the main indication for induction of labor, comprising 65% of cases. The next most frequent cause was pregnancy-induced hypertension. These women either had severe pre-eclampsia or eclampsia, where continuation of pregnancy was life-threatening for the mother, and the fetus was not salvageable without severe morbidity. These were performed after detailed counselling was undertaken and informed consent was obtained from the parents. Life-threatening congenital anomalies constituted one-fifth of cases, and abruptio placentae, severe fetal growth restriction and non-immune hydrops constituted the remainder.

At <28 weeks’ gestation, nearly twice the number of women required labor induction than those who did not (42.5% vs 20%) (Table 7). This was due to the greater prevalence of early-onset pre-eclampsia and lethal congenital anomalies in this group. The maximum number of women in the spontaneous labor group were in the gestational age of 28 to 33+6 weeks (Table 7).

**Fetal characteristics:** Of the stillborn babies, 51.4% (n=125) weighed less than 1000 g; 34.1% (n=83 babies) weighed between 1001 and 2500 g. Thirty-five (14.4%) babies weighed more than 2500 g (Table 4). Sixty-nine babies (28.3%) were found to be growth restricted based on WHO growth charts.

Lethal congenital anomalies were diagnosed in 45 (18.2%) cases. The incidence of congenital anomalies was significantly greater among the booked patients than the un-booked patients (24.7% vs 2.3%, p=0.001). Almost an equal proportion of babies were fresh and macerated stillbirths (Table 8).

### ReCoDe classification

The ReCoDe classification for our cohort of patients is tabulated below (Table 9). We were able to successfully classify 84.2% of stillbirths, leaving 15.78% unclassified. 

Labor was induced in 43 pregnancies due to the presence of major congenital anomalies identified in antenatal scans, resulting in stillbirths. Two stillborn babies referred from elsewhere as IUD, were found to have congenital anomalies at birth. The majority of these (n=32, 64%) had multiple major structural anomalies involving two or more systems. There were six cases of open neural tube defects; four had cardiac anomalies, the most common lesion being hypoplastic left heart. Out of four newborn diagnosed as having genitourinary abnormalities, one had bilateral renal agenesis and the rest had infantile polycystic kidneys with anhydramnios. Four cases had lethal skeletal dysplasia. All scans were performed by a sonographer accredited by the FMF, United Kingdom. Most parents, however, did not give consent for fetal autopsy.

Under class A (fetal causes), the maximum cases were classified under fetal growth restriction (n=64, 26.3%). We were able to identify a secondary code in all cases of fetal growth restriction. Uteroplacental insufficiency, by definition, was associated with all cases of fetal growth restriction, either as a secondary or tertiary code. Fetal growth restriction was diagnosed when the EFW measured on scan, was less than the 10th centile, based on WHO sex-specific growth curves. Using customized growth charts would have been a better option; however, we did not have access to these. Moreover, the authors of the study concluded that the lower centiles (10^th^, 5^th^, and 2.5^th^) of a pooled study were more universally applicable than the upper centiles (90^th^, 95^th^, and 97.5^th^), which may vary according to the population being studied (9). 

Cord accidents accounted for only 0.4% of cases, whereas placental causes constituted 12.5% (n=31). Among placental causes, the major part of cases was due to abruption (27/31; 87%). Abruption accounted for 10.9% of overall causes of stillbirths. Among the cases of abruption, 14.8% (4/27) had uteroplacental insufficiency. Out of a total of 27 cases of abruption, 29.6% (n=8) were associated with pregnancy-induced hypertension.

Sixteen women had hypertensive disorders of pregnancy as a primary code, this increased to 85 women (34.97%) when secondary and tertiary codes were added. Associated medical conditions were present in 46.5% (113/243 pregnancies). Out of these, 59.29% (n=67) had diabetes. Obstetric cholestasis was diagnosed in 1.76% (2/113) of cases, and APLA was diagnosed in 5.3% of cases (6/113). More than one medical comorbidity was detected in most women, the combination of diabetes and hypertension being the most common.

There were two cases of intrapartum stillbirths. One woman was referred from elsewhere in active labor at 38 weeks with cord prolapse and intrauterine demise and delivered a fresh stillborn baby. Another one was a gravida 4 para 3 referred from elsewhere with obstructed labor and IUD. At laparotomy, there was a uterine rent in the posterior uterine wall with the baby in the peritoneal cavity.

Chorioamnionitis was the assigned primary code in two cases (0.8%), whereas no relevant condition was identified in 39 cases (15.7%).

We were able to identify a secondary code in 49.3% of cases (122/247) (Table 9). The most common secondary code identified was uteroplacental insufficiency 40.16% (49/122). The second most common was pregnancy-induced hypertension 25.4% (31/122).

Tertiary codes were identified in 50 cases (20.2%). Pregnancy-induced hypertension made up 52% of these (26/50) (Table 10).

### Twins

There were eleven sets of twins included in our study, of which three were monochorionic diamniotic (MCDA) and eight were dichorionic diamniotic (DCDA) (Table 11). Of these 22 babies, 16 were stillborn. Five of these deaths occurred in relation to hypertensive disease, other causes included diabetes mellitus, lethal anomaly, abruption. Significant growth discordance was seen in 4 sets of twins, two DCDA and two MCDA twins. Of the two MCDA twins, the causes of discordant growth were twin-to-twin transfusion syndrome (TTTS) and selective fetal growth restriction. Two sets of twins had lethal congenital anomalies in one of the twins. In the DCDA twin, selective fetal reduction was performed for the anencephalic fetus, leading to macerated stillbirth. In the MCDA twin, selective cord occlusion was performed for the anomalous twin, resulting in stillbirth.

## Discussion

The incidence of stillbirths at our center for 2017 was found as 16.8 per 1000 births. This is lower than that quoted for India (23/1000 live births), but higher than that for the state of Tamil Nadu (7/1000, for 2014). Our center, being a tertiary level perinatal center, often gets referrals of high-risk pregnancies in which the fetus is already compromised.

Using the ReCoDe classification, we were able to classify 84.2% of stillbirths, leaving 15.78% unclassified. This is similar to the findings of the original authors who could not classify15.2% of cases (3). 

This system of classification has relevance in the developing world where there is often little information available at the time of delivery. Post mortem of the dead fetus and placental biopsy though routinely conducted in the developed world as a part of the evaluation, is often not performed in third world countries due to unwilling parents and lack of technical expertise. The ReCoDe classification system may be a great boon for elucidating causes of stillbirth in low resource settings because it does not depend upon the above-mentioned tests. 

The most common cause of stillbirths in our cohort was fetal growth restriction (25.9%). All these cases, by definition, were associated with uteroplacental insufficiency. We used the presence of two or more clinical indicators of maternal vascular malperfusion such as FGR, oligohydramnios, and abnormal pulsed-wave Doppler to define cases of uteroplacental insufficiency. Although such cases should ideally be corroborated with findings on placental histopathology, this could not be done due to a lack of expertise and /or unwilling parents. More than half of the cases of uteroplacental insufficiency were also associated with at least one other medical comorbidity such as hypertension, diabetes or APLA. Only half of these cases (n=51; 55.4%) were un-booked pregnancies, the rest had pregnancies supervised in our hospital. Three-quarters of stillbirths due to fetal growth restriction occurred in the third trimester, emphasizing the need for close antenatal surveillance during this period.

The above findings emphasize the need for close antenatal surveillance of pregnancies with medical comorbidities and uteroplacental insufficiency. Optimal treatment of underlying medical conditions may lead to a decrease in the incidence of fetal growth restriction and other associated complications. When diagnosed, optimal management and timely delivery of growth restricted fetuses is of utmost importance. Babies that are growth restricted have less energy reserve, tolerate labor less readily, and are thus more prone to ante/intrapartum asphyxia (3).

Four cases of abruption were associated with fetal growth restriction (and hence some degree of uteroplacental insufficiency). This was not statistically significant but in line with the theory that abruptio placenta is a manifestation of uteroplacental insufficiency (11). Of the six women with bicornuate uterus, 50% had placental abruption. Uterine anomalies are known to have a higher preponderance to abruption (12).

Associated medical conditions were present in 46.5% (n=113 pregnancies). Most women with medical disorders had at least two comorbidities, the most common combination being chronic hypertension and diabetes mellitus. Of these, the most common medical condition was diabetes mellitus, seen in 59.2% (67/113) of cases. The contribution of diabetes mellitus to overall stillbirths was 27.5% (67/243). This is similar to findings by Ajini et al. (13) who found 14.6% had diabetes. This is not surprising, given that India has the highest number of cases with diabetes mellitus in the world (31.7 million cases in 2000) (14). Undiagnosed/uncontrolled diabetes mellitus in pregnancy can contribute to stillbirths in many ways: higher incidence of congenital anomalies, severe growth restriction due to underlying vasculopathy and or/associated hypertension, macrosomia, and sudden intra-uterine demise at term. Thus, the need for universal screening and optimum control of diabetes in pregnancy is particularly relevant in India.

Hypertensive disorders encompassing both chronic hypertension and pregnancy-induced hypertension were found in 25.5% (62/243) pregnancies, making up 54.8% (62/113) of medical comorbidities. Gardosi et al. (3) found an incidence of hypertensive disease of only 0.8%. Ajini et al. (13) found an incidence of 27.6%, and Rajagopal et al. (15) found an incidence of 28%. Notably, the incidence of severe hypertensive disorders (severe pre-eclampsia/eclampsia/HELLP syndrome) among booked patients was nearly half. 

In our practice, we often find un-booked mothers seeking medical attention for severe hypertensive disorders at a stage where the disease pathophysiology is so advanced as to endanger the life of the mother, with accompanying severe growth restriction. In such cases, we find induction of labor, unfortunately, to be the only recourse. With regular antenatal visits, gestational hypertension is likely to be picked up at an early stage with optimal treatment, monitoring and timely delivery which, in turn, is likely to prevent stillbirths secondary to utero placental insufficiency. 

Preterm births resulting in stillbirths were seen in 2.42% cases (6/247). Most of these were referred from elsewhere with preterm premature rupture of membranes and/or preterm labor. These were classified under I1 (no relevant condition identified) in the ReCode classification, simply for the lack of a relevant category. Currently, we do not offer cervical length screening in low-risk asymptomatic women. In women with a previous history of mid-trimester loss, it would be prudent to monitor cervical length on serial scans and support pregnancy with progestogens. Cervical stitch should be considered when cervical length is ≤2.5 cm in singleton pregnancies (16).

Close monitoring of pregnancies with twins is very important. In monochorionic twins, two-weekly scans to screen for TTTS between 16-26 weeks is of paramount importance. Early diagnosis of TTTS will help by timely interventions such as laser therapy, which can salvage the pregnancy (17). In dichorionic twins, serial growth scans at 2-3-week intervals to pick up discordant growth between the twins is important. Frequent monitoring of such pregnancies will allow timely delivery of both babies before any mishap in the form of intrauterine fetal demise occurs.

### Study limitations

The retrospective nature of our study made us reliant on data collection by the treating physician at the time. There were no controls, which would have enabled better qualitative analysis. Fetal autopsy was not performed routinely, neither was placental histopathology. 

We used clinical indicators of uteroplacental insufficiency rather than placental biopsy findings because the latter is not routinely conducted in our hospital due to either a lack of expertise or unwilling parents. This, however, is unlikely to exaggerate the contribution of underlying uteroplacental insufficiency towards occurrence of stillbirths because we used strict clinical criteria, usually more than one, for defining such cases.

A large number of stillbirths in our country can be better defined by using the ReCode classification. The ReCoDe classification enabled us to classify 84.2% of these cases. The largest number of cases were due to underlying uteroplacental insufficiency resulting in fetal growth restriction. A better understanding of etiopathogenesis will help improve health care facilities and development of clinical guidelines, which will work towards alleviating this problem. There was a significantly greater chance of having stillbirths secondary to hypertensive disorders in women whose pregnancies were unsupervised or poorly supervised. A more comprehensive antenatal care system, with emphasis on regular visits, may help to diagnose the antecedent causes of avoidable stillbirths and lessen the burden of stillbirth in our country.

## Figures and Tables

**Table 1:**
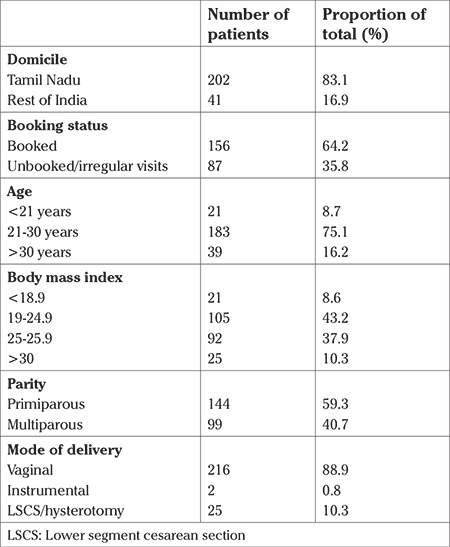
Maternal characteristics

**Table 2:**
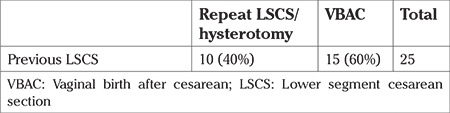
Mode of delivery for previous LSCS

**Table 3:**
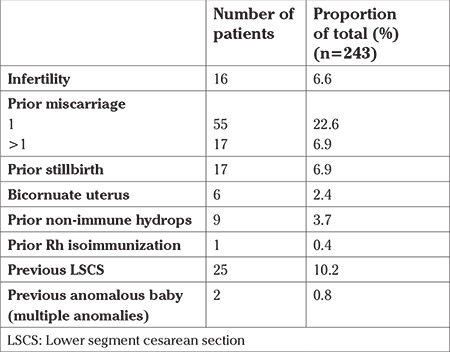
Obstetric risk factors

**Table 4:**
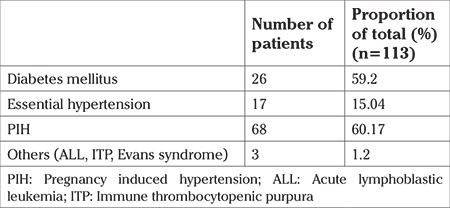
Medical risk factors

**Table 5:**
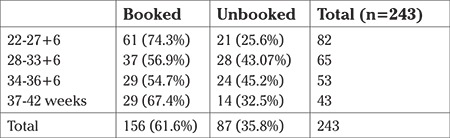
Gestational age at delivery

**Table 6:**
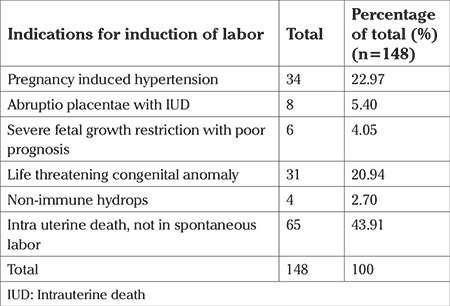
Indications for induction of labor

**Table 7:**
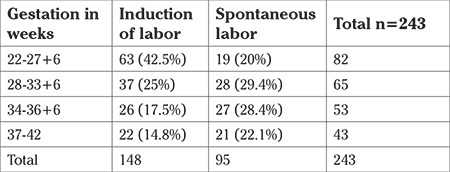
Induction of labor versus spontaneous labor in women with stillbirths at various gestational ages

**Table 8:**
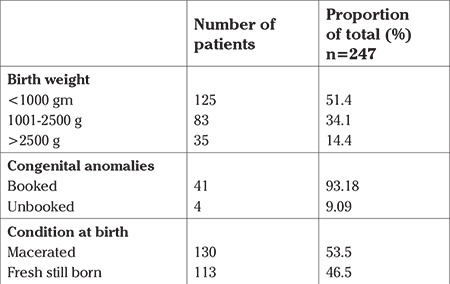
Fetal characteristics

**Table 9:**
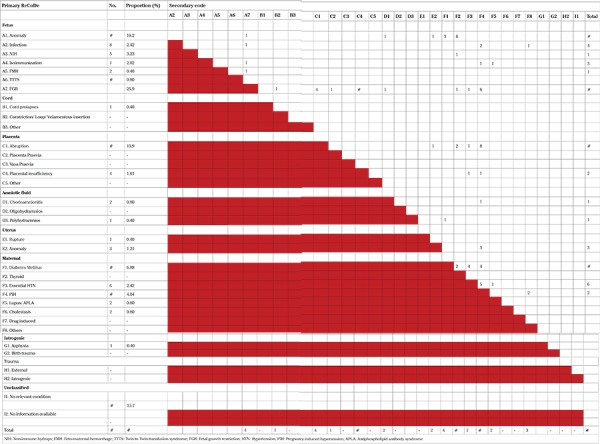
ReCoDe classification, primary and secondary code

**Table 10:**
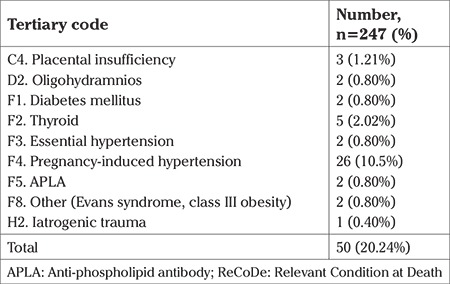
ReCoDe classification, tertiary code

**Table 11:**
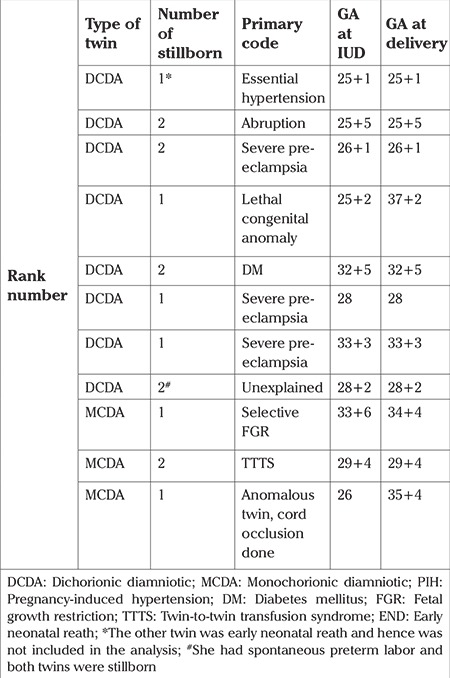
Twins
